# Carbonic anhydrase 3 increases during liver adipogenesis even in pre‐obesity, and its inhibitors reduce liver adipose accumulation

**DOI:** 10.1002/2211-5463.13376

**Published:** 2022-02-10

**Authors:** Hiroyuki Yamamoto, Naoto Uramaru, Azusa Kawashima, Toshiyuki Higuchi

**Affiliations:** ^1^ Department of Microbiology and Molecular Cell Biology Nihon Pharmaceutical University Kitaadachi‐gun Japan; ^2^ Department of Health Biosciences Nihon Pharmaceutical University Kitaadachi‐gun Japan

**Keywords:** carbonic anhydrase 3, fatty liver, lipid accumulation, peroxisome proliferator‐activated receptor gamma, pre‐obesity

## Abstract

The abnormal lipid metabolism in the liver that occurs after high caloric intake is the main cause of nonalcoholic fatty liver disease (NAFLD). Differences between samples from healthy livers and livers from individuals with NAFLD indicate that changes in liver function occur during disease progression. Here, we examined changes in protein expression in a fatty liver model in the early stages of obesity to identify potential alterations in function. The proteins expressed in the liver tissue of pre‐obese rats were separated *via* SDS/PAGE and stained with Coomassie brilliant blue‐G250. Peptide mass fingerprinting indicated an increase in the expression of carbonic anhydrase 3 (CA3) relative to controls. Western blotting analysis confirmed the increase in CA3 expression, even in an early fat‐accumulation state in which excessive weight gain had not yet occurred. In human hepatoma HepG2 cells, fat accumulation induced with oleic acid also resulted in increased CA3 expression. When the cells were in a state of fat accumulation, treating them with the CA3 inhibitors acetazolamide (ACTZ) or 6‐ethoxyzolamide (ETZ) suppressed fat accumulation, but only ETZ somewhat reduced the fat‐induced upregulation of CA3 expression. Expression of CA3 was therefore upregulated in response to the consumption of a high‐fat diet, even in the absence of an increase in body weight. The suppression of CA3 activity by ACTZ or ETZ reduced fat accumulation in hepatocytes, suggesting that CA3 is involved in the development of fatty liver.

## Graphical abstract

Here, we have shown that carbonic anhydrase 3 (CA3) expression was enhanced as a result of high‐fat diet consumption, even in the absence of an excessive increase in body weight. In addition, suppression of CA3 expression and/or activity by 6‐ethoxyzolamide (ETZ) or acetazolamide (ACTZ) reduced fat accumulation in hepatocytes, suggesting that CA3 is involved in the development of fatty liver.

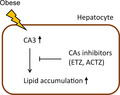

**Abbreviations**
ACTZ: acetazolamide
CA: carbonic anhydrase
CA3: carbonic anhydrase 3
ETZ: 6‐ethoxyzolamide
GAPDH: glyceraldehyde‐3‐phosphate dehydrogenase
NAFLD: nonalcoholic fatty liver disease
PMF: peptide mass fingerprinting
PPARγ: peroxisome proliferator‐activated receptor gamma
SDS/PAGE: sodium dodecyl sulfate/polyacrylamide gel electrophoresis
TFA: trifluoroacetic acid
α‐CHCA: α‐cyano‐4‐hydroxycinnamic acid

Obesity causes the enlargement of adipocytes, which results in abnormal adipocytokine secretion and increased fatty acid secretion. These effects reduce tissue insulin sensitivity and lead to insulin resistance, resulting in abnormal energy metabolism [1, 2]. These energy metabolism abnormalities then cause fat accumulation, which occurs not only in adipose tissue but also in the liver. This occurs because as glucose uptake decreases because of the reduced insulin sensitivity, blood glucose levels rise and insulin secretion increases. Large quantities of free fatty acids are released from the adipose tissue, which has reduced its insulin‐dependent inhibition of triglyceride degradation, and these free fatty acids are taken up by the liver [3]. Hyperinsulinemia promotes lipid synthesis in the liver and results in the local accumulation of fat, which is termed fatty liver disease. This condition is strongly associated with excessive alcohol consumption, but when it is caused by metabolic disorders, it is called nonalcoholic fatty liver disease (NAFLD), since the causative mechanism is not alcohol‐dependent [4]. NAFLD, which only causes deposition of fat in the liver, often progresses to nonalcoholic steatohepatitis (NASH), which causes fibrosis and increases the risk of cirrhosis and liver cancer, although the mechanistic details of the progression are unclear [5, 6, 7].

It has been established that the accumulation of fat in hepatocytes alters their expression of the proteins they produce [8]. Their expression of cytokines is altered by the effects of lipid accumulation on energy metabolism. That is, when fat accumulates in hepatocytes, as in adipocytes, adipokine expression changes; for example, adiponectin expression decreases and the expression of tumor necrosis factor‐alpha and leptin increases [9]. The expression of hepatokines such as sex hormone‐binding globulin, angiopoietin‐related growth factor, fibroblast growth factor (FGF) 19 and FGF21, fetuin A, fetuin B, retinol‐binding protein 4, and selenoprotein P is also affected [10, 11]. Thus, fat accumulation in hepatocytes affects the expression of proteins involved in a range of functions, including various metabolites, energy storage molecules, digestive enzymes, and growth factors.

Carbonic anhydrases promote fatty acid synthesis in adipocytes and the liver [12, 13, 14]. In addition, the expression of carbonic anhydrase 3 (CA3) enhances fat accumulation during the process of differentiation from preadipocytes to adipocytes. However, it has been shown that a CA inhibitor, 6‐ethoxyzolamide (ETZ), can suppress fat accumulation, and it also downregulates peroxisome proliferator‐activated receptor gamma (PPARγ) activation, which inhibits the differentiation of preadipocytes to adipocytes [15, 16].

Previous studies on the effects of adipose tissue‐derived adipocytokines have been often performed using fatty livers from obese animal models, so it is unclear whether the same mechanisms are active in cases of fatty liver without obesity. In this study, we investigate changes in protein expression in early‐stage fatty liver without weight gain, induced by high‐fat diet consumption. Our objective is to identify proteins whose expression is altered and determine how their expression is regulated.

## Materials and methods

### Materials

We obtained ETZ and acetazolamide (ACTZ) from Sigma‐Aldrich (St. Louis, MO, USA). We purchased the high‐fat diet HFD‐60 from Oriental Yeast (Tokyo, Japan). All other chemicals used were of reagent grade.

### Cell culture

The human hepatocellular carcinoma cell line HepG2 was supplied by the Health Science Research Resources Bank. We cultured the cells in Dulbecco’s modified Eagle’s medium (DMEM; Fujifilm Wako Pure Chemical, Osaka, Japan) supplemented with 10% fetal bovine serum (FBS; Hyclone, London, UK) under humidified air containing 5% CO_2_ at 37 °C. For lipid accumulation, we placed 70–80% confluent cells in DMEM containing 10% FBS and 0.5 mm oleic acid. We treated the cells for two days with ETZ or ACTZ, which we added to the culture medium at a final concentration of 100 or 200 μm, to examine their effects on lipid accumulation and protein expression.

### Animals

We purchased male Wistar rats from Japan SLC (Shizuoka, Japan). All rats were housed in specific pathogen‐free facilities and had access to tap water and food ad libitum. Lights were automatically turned on at 8:00 and off at 20:00. All animal experiments were approved by the Animal Experimentation Ethics Committee of Nihon Pharmaceutical University and studies were performed under Nihon Pharmaceutical University guidelines for the use of animals.

In preparation for the experiments, all rats were fed a normal diet until they reached six weeks of age. Thereafter, they were fed a normal diet (normal diet group) or a high‐fat diet (high‐fat diet group) for four weeks. The rats were sacrificed at 10 weeks of age and the liver was extracted. We prepared liver homogenate in 10 mm Na, K‐phosphate buffer (pH 7.4) containing 1.15 m KCl. The livers and the homogenate samples were frozen immediately and stored at −80 °C. We used the homogenate to analyze protein expression in a western blot analysis.

### Histochemistry

We dissected the livers and fixed them with 4% paraformaldehyde (PFA) in phosphate‐buffered saline, which we then exchanged with 20% sucrose (overnight) and 30% sucrose (overnight), before embedding them in O.C.T. compound (Sakura Seiki, Tokyo, Japan). Sections were prepared using a cryostat and dried overnight at room temperature.

### Lipid staining

We stained the HepG2 cells and liver sections for lipids using Oil Red O [17]. The amount of dye present was determined based on absorbance at a wavelength of 540 nm.

### Protein quantification assay

We measured the protein content using the bicinchoninic acid method, with bovine serum albumin as the standard.

### Sodium dodecyl sulfate/polyacrylamide gel electrophoresis (SDS/PAGE)

We reduced the cell extracts and liver homogenate with mercaptoethanol at 95 °C for 10 min. The protein samples were separated on a 12% polyacrylamide gel [18], and we stained the proteins with Coomassie brilliant blue‐G250 staining solution [19].

### Protein identification *via* peptide mass fingerprinting analysis

The bands detected using Coomassie brilliant blue‐G250 were collected and incubated with 30% acetonitrile in 0.1% trifluoroacetic acid until the color disappeared. We then modified the proteins through alkylation with 10 mm dithiothreitol and 50 mm monoiodoacetic acid. The modified proteins were digested overnight with 10 ng·μL^−1^ N‐tosyl‐l‐phenylalanine chloromethyl ketone‐trypsin (Sigma‐Aldrich) in 100 mm ammonium bicarbonate at 37 °C. For desalting, we loaded the digested proteins into ZipTip C18 pipette tips (Millipore, Billerica, MA, USA) and eluted them with 0.1% trifluoroacetic acid (TFA)/acetonitrile (1 : 2, v/v). Next, we performed mass spectrometry on a Bruker Autoflex (Bruker Daltonics, Bremen, Germany). We mixed the digested samples with an equal volume of saturated α‐cyano‐4‐hydroxycinnamic acid (α‐CHCA) (Sigma‐Aldrich) in 0.1% TFA/acetonitrile (1 : 2, v/v) and applied them to a target plate. We obtained the spectra in the positive mode and analyzed them with FLEXAnalysis (Bruker Daltonics). We used the Mascot program (Matrix Science, London, UK) to conduct database searches.

### Western blotting analysis

We reduced the cell extracts and liver homogenate samples with mercaptoethanol at 95 °C for 10 min. We loaded the samples at 10 μg of protein per lane and then separated them on a 12% polyacrylamide gel. Following the electrophoresis, we blotted the proteins on a nitrocellulose membrane (ClearTrans Nitrocellulose Membrane; Fujifilm Wako Pure Chemical) in a semidry blotting system (NA‐1512S; Nihon Eido, Tokyo, Japan) [20]. The nitrocellulose membranes were blocked with 2% skim milk. We then incubated the membranes with rabbit anti‐CA3 antibody (1 : 8000; Proteintech, Rosemont, IL, USA), rabbit anti‐catalase antibody (1 : 10,000; produced by our laboratory), mouse anti‐β actin monoclonal antibody (1 : 10,000; Fujifilm Wako Pure Chemical), or mouse anti‐glyceraldehyde‐3‐phosphate dehydrogenase monoclonal antibody (1 : 5000; Fujifilm Wako Pure Chemical). This was followed by incubation with horseradish peroxidase (HRP)‐conjugated goat anti‐rabbit immunoglobulin G (IgG) antibody (1 : 10,000; SeraCare, Milford, MA, USA) or HRP‐conjugated goat anti‐mouse IgG antibody (1 : 2500; Biosource, Camarillo, CA, USA). Finally, we visualized the protein bands using ImmunoStar LD or ImmunoStar Zeta (both Fujifilm Wako Pure Chemical) with a LuminoGraph (Atto, Tokyo, Japan). We subjected the bands to densitometric analysis using imagej (National Institutes of Health, Bethesda, MD, USA).

### Statistical analysis

Data are presented as means ± standard deviation (SD). We analyzed the results *via* Mann–Whitney *U‐*tests conducted in R (R Development Core Team), and we considered *P*‐values < 0.05 to indicate statistical significance.

## Results

### Alteration of protein expression in fatty liver tissue was induced by a high‐fat diet

**Fig. 1:**
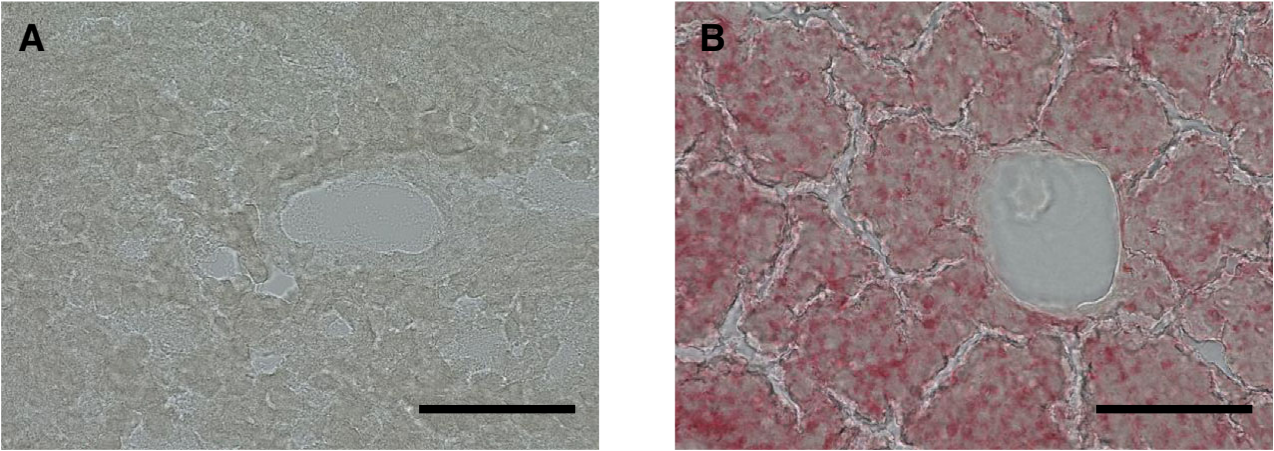
Fat accumulation in the liver of rats that consumed a high‐fat diet. Frozen liver tissue sections were stained for lipids using Oil Red O. (A) Normal diet, rats aged 10 weeks. (B) High‐fat diet, rats aged 10 weeks and fed a high‐fat diet for four weeks (weeks 7–10). Magnification: 200×; scale bars: 100 μm.

**Fig. 2:**
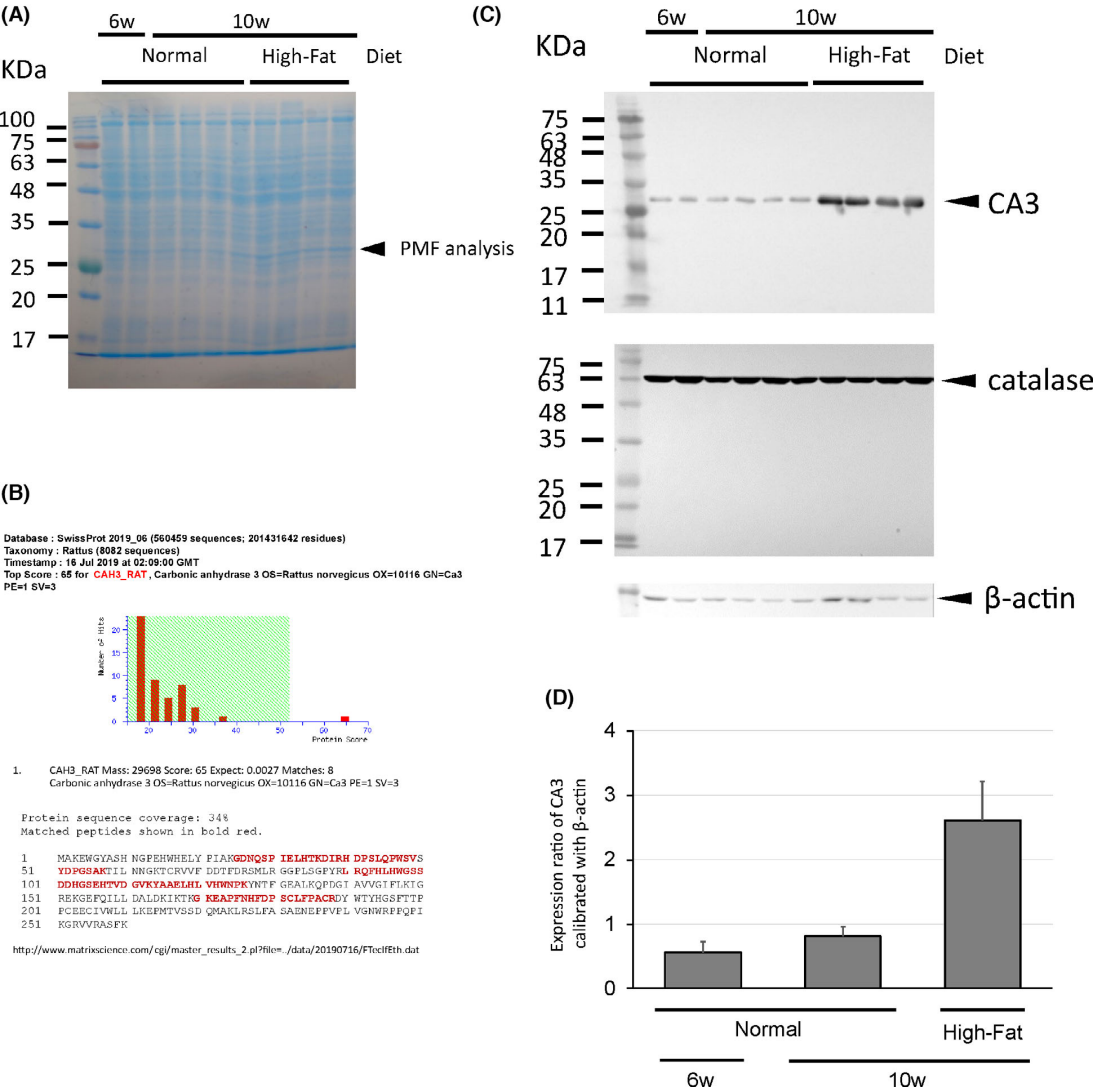
Expression of CA3 in fatty liver induced by the consumption of a high‐fat diet. (A) Changes in protein expression after consumption of a high‐fat diet for four weeks (SDS/PAGE analysis). (B) The search results of the Mascot program. The fingerprinting analysis yielded a match for rat carbonic anhydrase 3, with a significant score of 65 (*P* = 0.0027). The sequence coverage was 34%. (C) Western blot analysis for the expression of CA3, catalase, and β‐actin in the liver. (D) Catalase and β‐actin were used as controls. The CA3 band density was measured using imagej, calibrated against β‐actin. Data presented are mean ± SD density (*n* = 4 or 8).

At six weeks, the body weight of the rats was 134 ± 5.3 g. At 10 weeks, body weight had increased to 257 ± 5.8 g in the high‐fat diet group and 248 ± 10.3 g in the normal diet group. The difference in body weight between the two groups was not significant. We analyzed fat accumulation in the liver histochemically and found numerous fat droplets in liver tissue sections from rats fed the high‐fat diet (Fig. 1B). In contrast, there were few fat droplets in sections from the normal diet group (Fig. 1A). This suggested that consumption of a high‐fat diet for four weeks enhanced fat accumulation in hepatocytes without increasing body weight. The expression of most proteins did not differ between the groups (Fig. 2A). However, at 10 weeks, there was an increase in the expression of one protein with a size of approximately 27 kDa in the high‐fat diet group, but not in the normal diet group. We therefore performed peptide mass fingerprinting. The fingerprinting analysis yielded a match for rat CA3 (Fig. 2B; Table 1), which was in the NCBI database under accession no. NP_062165.2. The sequence coverage was 34%. We conducted a western blotting analysis to verify the expression of the CA3 protein (Fig. 2C,D). The results of this analysis were consistent with those of SDS/PAGE, confirming that CA3 expression increased only in the high‐fat diet group and did not change from 6 to 10 weeks in rats fed the control diet. Moreover, there was no significant change in the expression of catalase (Fig. 2C), another protein expressed in the liver, when β‐actin was used as an internal standard. This suggested that the expression of CA3 increased in fatty liver tissue specifically in response to the consumption of a high‐fat diet.

**Table 1:** Peptide fingerprinting of CA3 from liver tissue from rats fed a high‐fat diet. The sequence of rat CA3 is in the top row.

Measured mass	Position	Sequence	Expected mass
1337.813	25–36	GDNQSPIELHTK	1336.8057
2356.079	37–57	DIRHDPSLQPWSVSYDPGSAK	2355.0717
1971.299	40–57	HDPSLQPWSVSYDPGSAK	1970.2917
2744.473	90–113	LRQFHLHWGSSDDHGSEHTVDGVK	2743.4657
2475.967	92–113	QFHLHWGSSDDHGSEHTVDGVK	2474.9597
1577.459	114–126	YAAELHLVHWNPK	1576.4517
2250.887	170–188	GKEAPFNHFDPSCLFPACR	2249.8797
2065.602	172–188	EAPFNHFDPSCLFPACR	2064.5947

### Induction of CA3 expression by fat accumulation in HepG2 cells and effect of CA3 on fat accumulation

**Fig. 3:**
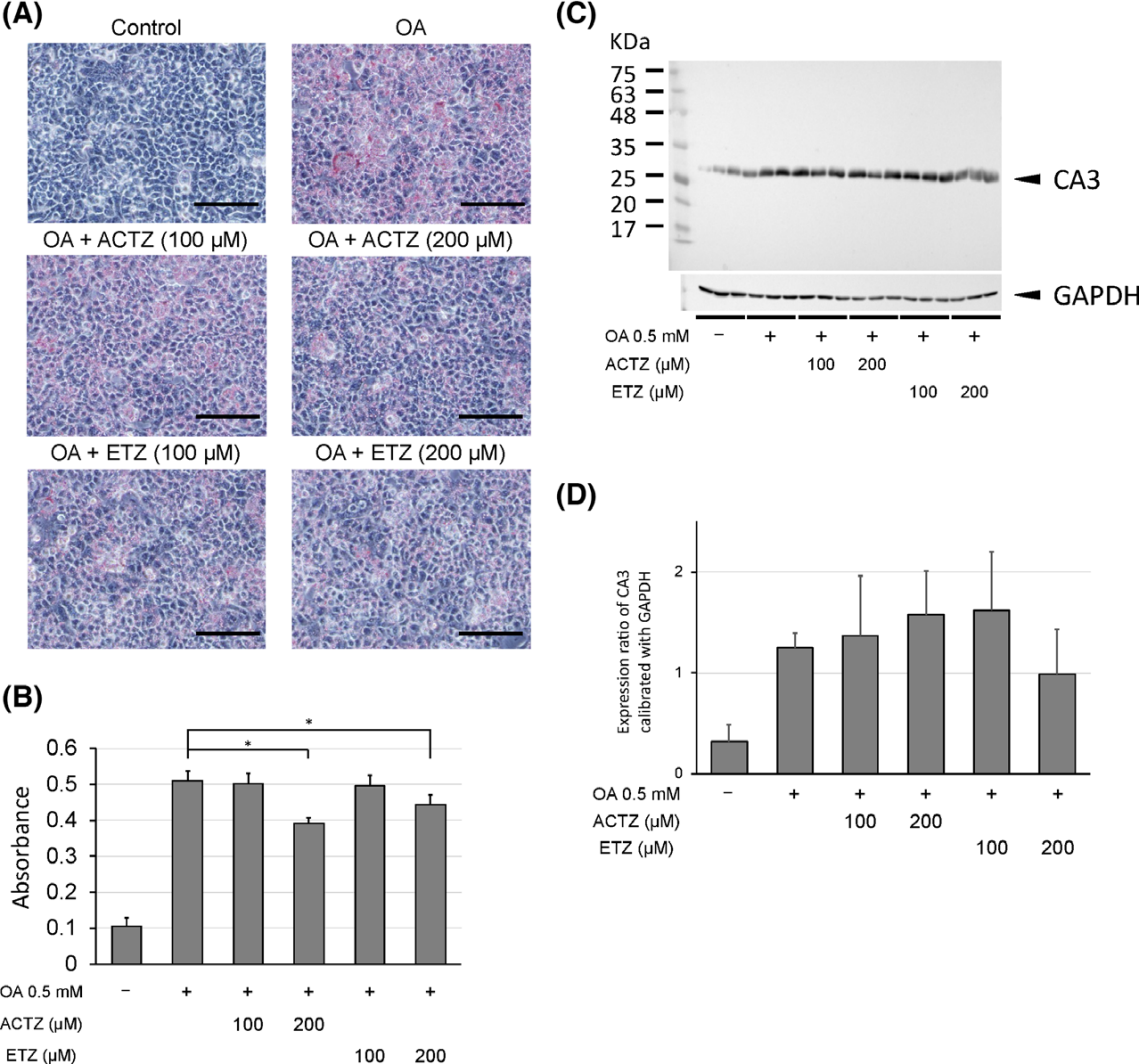
Fat accumulation and carbonic anhydrase 3 (CA3) expression in human hepatoma HepG2 cells induced by culturing in medium containing oleic acid (OA). We tested the effects of acetazolamide (ACTZ) or ethoxyzolamide (ETZ), both CA3 inhibitors, on fat accumulation and CA3 expression. (A, B) Lipids in the HepG2 cells were stained using Oil Red O (A) and quantitatively analyzed by measuring dye absorbance at 540 nm (B). Magnification: 200×; scale bars: 500 μm. The data presented are mean ± SD (*n* = 6) absorbance relative to the control (no CA3 inhibitors). * *P* < 0.05, Mann–Whitney *U‐*tests compared with OA‐induced fat accumulation. (C) Western blot analysis of CA3 and GAPDH expression in HepG2 cells. GAPDH was used as an internal standard. (D) Band density was measured using imagej, calibrated against GAPDH. Data presented are mean ± SD density (*n* = 6).

Culturing HepG2 cells with oleic acid resulted in numerous cells containing red‐stained fat droplets, indicating fat accumulation (Fig. 3A,B). There was no fat accumulation in the cells cultured without oleic acid. Adding a CA3 inhibitor (ACTZ or ETZ) significantly reduced the number of fat droplets in the cells. Including oleic acid in the medium to induce fat accumulation also caused higher CA3 expression in the cell lysate (Fig. 3C,D). This upregulation of CA3 was reduced slightly by adding ETZ, but not by adding ACTZ.

## Discussion

In this study, we found that the expression of CA3 was upregulated in fatty liver tissue from rats that consumed a high‐fat diet but did not gain excess body weight. CA3 expression was not affected by aging alone (in the age range 6–10 weeks) in rats fed a normal diet.

Carbonic anhydrases (CAs) catalyze the hydration of carbon dioxide to bicarbonate and protons. They are abundant in erythrocytes, gastric acid‐secreting cells, and renal tubules and are involved in maintaining the pH of body fluids. Protons and carbonic acid, which are regulated by CAs, are involved in numerous metabolic processes, such as urine production, respiration, transport of carbon dioxide and ions, bone resorption, gluconeogenesis, body fluid production, and fatty acid synthesis [21, 22, 23].

The CAs are related to lipogenesis because of the production of bicarbonate in hepatocytes. Bicarbonate serves as a substrate for acetyl‐CoA carboxylase when it catalyzes the carboxylation of acetyl‐CoA to malonyl‐CoA [24]. CA3 is abundant in skeletal muscle, adipocytes, and the liver [25, 26]. CA3 expression levels are affected by several factors, such as obesity (fat accumulation), insulin, and adipokines. A mouse model study demonstrated that obesity reduces CA3 mRNA expression in adipose tissue, whereas there was no change in muscle or liver tissue [27]. CA3 expression in adipose tissue is upregulated by insulin and downregulated by leptin [28]. In addition, insulin depletion in streptozotocin‐induced diabetic rats reduced the expression of CA3 in adipose tissue and the liver [29, 30].

Our finding that CA3 expression was upregulated in the liver during fat accumulation was inconsistent with the results reported by Stanton et al. [27]. We have two hypotheses to explain this discrepancy. First, the tissue we used was from the fatty livers of rats that had not yet gained excess weight, which may represent a different stage of NAFLD. The increased CA3 expression we observed likely promotes fat accumulation in hepatocytes by acting on CO_2_ generated from the citric acid cycle and other sources, which is consistent with Stanton et al. [27]. Second, Stanton et al. [27] assessed CA3 mRNA levels, whereas we evaluated protein levels. This suggests that CA3 expression in the liver may be more strongly regulated by the post‐translational CA3 degradation system than by mRNA transcription. Therefore, regulation of CA expression in the fatty liver of an individual with normal body weight may differ from that in an obese individual. We also showed that fat accumulation enhanced CA3 expression in HepG2 cells, supporting the hypothesis that CA3 expression in the liver is regulated by a different mechanism than in adipose tissue. Further experiments are necessary to test these hypotheses.

Recent studies have investigated the function of CA3 in CA3‐deficient mice, demonstrating that the enzyme deficiency may impair mitochondrial adenosine triphosphate synthesis in muscle tissue and fatty acid metabolism, but does not affect growth or lifespan [25, 31]. In particular, the impairment of fatty acid metabolism resulting from CA3 deficiency supports the idea that CA3 is necessary for fatty acid synthesis, which produces bicarbonate ions, a substrate for pyruvate carboxylase and acetyl‐CoA carboxylase [13].

CA3 expression is enhanced during the process of differentiation from preadipocytes to adipocytes, and PPARγ agonist stimulates the differentiation into adipocytes with increased insulin sensitivity, fat accumulation, and enhanced CA3 expression [15]. However, when ETZ is added to PPARγ agonist‐stimulated cells, it suppresses the CA3 expression and differentiation into adipocytes that occur with fat accumulation, although ACTZ, also a CA inhibitor, has no effect on fat accumulation in adipocytes [15]. Here, we investigated the expression of CA3 in HepG2 cells after treatment ACTZ and ETZ. The addition of ACTZ suppressed fat accumulation but did not affect CA3 expression in cells cultured with oleic acid. However, treatment with ETZ suppressed both fat accumulation and CA3 expression. Thus, the role of CA3 in fat accumulation in adipocytes and hepatocytes is different, since ACTZ, which inhibits CA3 but does not affect its expression, did not affect fat accumulation in adipocytes, whereas it suppressed it in hepatocytes. In addition, CA3 expression is reduced in alcohol‐induced liver injury [32], and the accumulation of fat in hepatocytes increases when alcoholic liver injury continues. This suggests that the mechanism of fat accumulation differs between alcoholic and nonalcoholic hepatotoxicity. However, further research is required to confirm this and to elucidate the mechanism of fat accumulation in hepatocytes.

In conclusion, we have shown that CA3 expression was enhanced as a result of high‐fat diet consumption, even in the absence of an excessive increase in body weight. In addition, suppression of CA3 expression and/or activity by ETZ or ACTZ reduced fat accumulation in hepatocytes, suggesting that CA3 is involved in the development of fatty liver.

## Conflict of interest

The authors declare no conflict of interest.

## Author contributions

HY, TH, and NU contributed to the experimental design. HY, TH, NU, and AK performed the experiment. HY and TH wrote the manuscript.

## Data Availability

The data that support the findings of this study are available from the corresponding author upon reasonable request.
